# Assessing the Clinical Utility of Antinuclear Antibody Titer Dilutions, Antinuclear Antibody Staining Patterns, and Other Common Laboratory Tests in Juvenile Idiopathic Arthritis

**DOI:** 10.7759/cureus.76648

**Published:** 2024-12-30

**Authors:** Willie S Van Heerde, Michael Jutovsky

**Affiliations:** 1 Medicine, Saint James School of Medicine, Amos Vale, VCT; 2 Family Medicine, MJ Medical Group, Chicago, USA

**Keywords:** ana hep-2 positivity, antibody titer, antinuclear antibody testing, juvenile idiopathic arthritis(jia), musculoskeletal pain

## Abstract

Chronic musculoskeletal pain in pediatric patients can be challenging to diagnose, particularly in the absence of overt signs of autoimmune disease, as these episodes can manifest episodically. We present a case of a 14-year-old female patient with a two-year history of episodic “bone pain,” morning stiffness, and infrequent fever and fatigue. Laboratory testing revealed an antinuclear antibody (ANA) titer of 1:1280 with a nuclear homogeneous pattern and a mildly elevated erythrocyte sedimentation rate (ESR). Although the patient did not meet the criteria for juvenile idiopathic arthritis (JIA), her laboratory findings and irregular musculoskeletal pain prompted the review of literature on the diagnostic value of ANA titers, ANA staining patterns, and other common lab tests in JIA.

## Introduction

Diagnosing juvenile idiopathic arthritis (JIA), formerly known as juvenile rheumatoid arthritis, poses significant challenges due to its diverse presentations and the overlap of its symptoms with other pediatric conditions. JIA is primarily characterized by chronic joint inflammation and may also include extra-articular symptoms such as rashes, fever, anemia, iritis, or uveitis.

Antinuclear antibody (ANA) testing is commonly included in the initial panel of tests when autoimmune conditions are suspected by a physician. ANAs are autoantibodies that target the nucleus of a cell and are commonly associated with autoimmune diseases [1]. However, low ANA titers can also be present in healthy children, complicating the diagnostic process, but the rate of false positives drops significantly as the titers increase [2]. Elevated ANA levels can suggest an increased risk of autoimmune disorders like systemic lupus erythematosus (SLE) [1].

In this report, we highlight the case of a 14-year-old female patient who presents with a two-year history of sporadic musculoskeletal pain and a markedly high ANA titer of 1:1280 with a homogeneous staining pattern. Such a high ANA titer is rare in the general pediatric population [1,3]. Additionally, the homogeneous staining pattern is notable for being commonly seen in JIA [4]. Although she is unlikely to have JIA, her test results and unusual musculoskeletal pain prompted a closer look at the value of high ANA titers in assessing JIA risk. This case underlines the importance of understanding the implications of elevated ANA levels in primary care settings.

## Case presentation

A 14-year-old female patient presented for an annual checkup with a complaint of episodic "bone pain" persisting for the past two years. The patient stated that she had not mentioned her pain or sought treatment until she experienced a marked exacerbation since her last annual checkup. The patient described the episodes as varying from half a day to two days and appearing randomly without any clear precipitating factors. The pain, which was usually bilateral primarily affected her ankles, knees, wrists, fingers, and shoulders. Each episode was characterized by morning stiffness that improved as the day progressed.

The patient rated the pain as a five out of ten on a typical episode, though it could escalate to a ten out of ten during severe episodes. When present, the episodes greatly impacted her ability to perform simple activities such as walking, operating door handles, and opening bottle screw caps. To manage the pain, she self-medicated with 400 mg of Advil during episodes but reported that this provided minimal relief.

The patient denied experiencing fever, rash, or other systemic symptoms typically associated with autoimmune or infectious processes during an episode. Her mother, who was present at the visit, denied any known history of autoimmune conditions on either side of the family. The patient’s BMI was within normal limits, and she has no known history of chronic conditions or previous medical diagnoses.

Physical examination

A comprehensive physical examination was conducted. Examination of the extremities revealed no visible deformities. The patient maintained a full range of motion in the ankles, knees, wrists, fingers, and shoulders. Observation of the nails showed no evidence of nail pitting or other deformities. Examination of the skin showed no lesions that would suggest a connective tissue disease. Examination of the neck showed no signs of lymphadenopathy or thyromegaly. At the time of the examination, the patient was not experiencing an acute episode of pain, which may have contributed to the absence of significant physical findings.

Laboratory investigation

Following the initial encounter, a laboratory workup was ordered to help identify what might be causing the patient’s symptoms (Table 1). The results were reviewed at the patient’s follow-up visit the next week. Of particular note, an abnormally elevated ANA titer with a homogeneous nuclear staining pattern suggested a potential autoimmune issue. At the same time, the combination of high alkaline phosphatase and low vitamin D prompted us to consider conditions that might explain the patient’s bone pain. This included considering metabolic bone diseases, autoimmune disorders, or other inflammatory problems.

**Table 1:** Table of laboratory results. ANA: antinuclear antibody, SLE: systemic lupus erythematosus, JIA: juvenile idiopathic arthritis, TSH: thyroid-stimulating hormone, CCP AB: cyclic citrullinated peptide antibodies, AC: anti-cell.

Parameter	Result	Reference value	
ANA titer	1:1280	<1:40	Negative
		1:40 - 1:80	Low antibody level
		>1:80	Elevated antibody level
ANA staining pattern	Nuclear, homogenous (AC-1)	AC-1 is associated with SLE, drug-induced lupus, and JIA	
Erythrocyte sedimentation rate	<28 mm/h	<=20 mm/h	
Rheumatoid factor	<14 IU/mL	<14 IU/mL	
CCP AB	<16	<20	Negative
		20 - 39	Weak positive
		40 - 59	Moderate positive
		>59	Strong positive
Alkaline phosphatase	183 U/L	51 - 179 U/L	
Vitamin D, 25-OH	16 ng/mL	30 - 100 ng/mL	
TSH with reflex to FT4	2.69 mlU/L	0.50 - 4.30 mIU/L	

Treatment plan and expected outcome

Based on the patient's clinical presentation, physical exam, and laboratory findings, the decision was made to refer the patient to a rheumatologist for further evaluation. The primary concern at this time was an unspecified connective tissue disease. During the interim, the patient and mother were educated on monitoring and recording new or worsening symptoms, such as prolonged morning stiffness and persistent joint swelling. The patient was advised to avoid overuse of over-the-counter pain medications, including acetaminophen or ibuprofen, and to report any worsening of symptoms prior to her specialist evaluation.

## Discussion

The patient’s presentation, marked by episodic musculoskeletal pain, morning stiffness, and an absence of systemic symptoms, raises intriguing questions about the potential correlation between her high ANA titer and the nuclear homogeneous staining pattern. Although her physical examination was unremarkable and lacked signs of active joint inflammation, the significantly elevated ANA titer (1:1280) should not be overlooked as most labs consider 1:80 or 1:160 as positive. The homogeneous pattern is often associated with autoimmune conditions, such as JIA, but the current absence of clear clinical evidence makes it difficult to establish any diagnosis. This case prompts consideration of whether the high ANA titer and staining pattern could represent an early marker of autoimmune disease or merely a nonspecific finding. Further longitudinal observation and research are needed to better understand the significance of these laboratory results in patients with subtle or intermittent symptoms.

Significance of ANA titers in juvenile idiopathic arthritis

ANA tests are still used extensively today as a rapid diagnostic tool for autoimmune conditions such as SLE. The primary issue with ANA is the rate of positivity in the healthy population. This rate of false positives can be decreased by increasing the cutoff dilution. By increasing the cutoff criterion, it is possible to turn 13.5% of false positives at 1:80 dilution into 6.3% at 1:160, 4% at 1:320, and finally 1.6% at 1:640 dilution [1]. Patients with JIA have been shown to possess higher ANA titers versus the general population, but a significant portion of those with JIA are likely to have a negative ANA result [2,4]. ANA titers were found to have a strong prognostic value for the risk of uveitis [2,5]. Elevated ANA titers above 1:160 were associated with worse clinical outcomes. Finally, the same study found that 68% of the patients had decreased levels on follow-up [4]. Elevated ANA titers are typically seen in specific subtypes such as oligoarticular, and likewise decreased in certain subtypes such as systemic JIA [4-6]. It is important to note that there exists no universally accepted ANA cutoff value between laboratories, and as such physicians must make sure to correctly interpret results. Due to the frequency of negative ANA tests in JIA, the fluctuations of titers within cases, and its inability to differentiate JIA from other autoimmune conditions, it has poor diagnostic potential by itself. However, it remains a valuable tool in confirmed cases, particularly for providing a prognosis and monitoring disease progression.

Potential significance of ANA staining pattern

The nuclear homogeneous staining pattern, which received international recognition from the International Consensus on ANA Patterns (ICAP) in 2014 [7], is now identified as AC-1. This categorization has been linked to SLE, autoimmune hepatitis, and JIA [8]. Subsequent research underscored that the homogeneous pattern is predominantly observed in autoimmune conditions associated with ANA, mirroring the challenges in differentiating these conditions from JIA [9]. Significantly, the study noted that this pattern is seldom found in healthy individuals, thereby aiding in minimizing false positive diagnoses. Furthermore, it was observed that some staining patterns, such as dense fine speckled, are less common in ANA-associated conditions, yet they still occur in cases of JIA and non-rheumatic diseases. This differentiation in staining patterns could improve diagnostic accuracy by reducing the differential diagnosis from three possibilities (other autoimmune conditions, JIA, or a healthy individual) to two, which could prove to be clinically valuable.

Another study reported that 50% of patients initially diagnosed with JIA showed a homogeneous staining pattern, but 38% of the ANA-positive patients exhibited a different pattern upon follow-up [4]. This suggests that the homogeneous pattern may not reliably predict an individual’s susceptibility to JIA outside of active episodes or other possible unknown criteria. The interpretation of staining patterns is inherently complex especially when considering the lack of a universally adopted system; this leads to potential error due to multiple factors such as lack of analyst skill, different laboratory methods or criteria, differences in opinion, and equipment limitations. These variables can significantly increase the likelihood of physicians receiving incorrect results [10-12]. Given the current gaps in research and the observed inconsistencies in stain identification, there is a compelling need for further studies. It is crucial to explore whether specific ANA staining patterns hold a more definitive association with JIA, which would be instrumental in enhancing diagnostic precision and patient management.

Significance of HLA-B27

An important discovery is the association between HLA-B27 and specific JIA subtypes, particularly enthesitis-related arthritis. HLA-B27 positivity is significantly associated with a higher occurrence of uveitis and a reduced likelihood of achieving disease remission [13,14]. One study found that 18.5% of uveitis cases occurred in HLA-B27-positive JIA patients, highlighting the clinical relevance of genetic markers in certain cases of JIA [6]. This association underscores the need for further investigation into genetic predispositions that could inform treatment strategies and predict disease outcomes.

Significance of rheumatoid factor

The rheumatoid factor (RF) test, while essential for distinguishing between RF-positive and RF-negative forms of JIA, also presents limitations. Only 10-15% of JIA cases test positive for RF, which diminishes its overall diagnostic utility [6,15]. The renaming of juvenile rheumatoid arthritis to juvenile idiopathic arthritis reflects this diagnostic complexity. By emphasizing the "idiopathic" nature of the condition, the name change helps avoid confusion, as approximately 85% of adult rheumatoid arthritis cases test positive for RF, a characteristic not shared by most JIA cases [15].

Miscellaneous findings in laboratory testing

Additional findings emphasize the variability and inconsistency in laboratory markers among JIA patients. A study conducted in Turkey identified factors associated with poorer treatment outcomes, such as thrombocytosis, delays in initiating treatment of more than six months, and the onset of symptoms before the age of five [16]. Furthermore, the unpredictable nature of standard lab results, such as white blood cell (WBC) counts, erythrocyte sedimentation rate (ESR), and C-reactive protein (CRP), complicates the diagnostic process. These markers can often remain within normal limits, even during active disease episodes with significant symptoms [6,15,16]. Consequently, clinicians must exercise caution and cannot solely rely on routine lab tests when diagnosing or managing JIA.

While researching the diagnostic landscape of JIA, we uncovered several findings that highlight the complexities of lab testing for this condition. An extensive study from Germany examined various autoantibodies commonly associated with autoimmune diseases but failed to establish a significant correlation between JIA and other autoimmune conditions, such as the antiphospholipid syndrome seen in SLE [17]. This lack of a discernible link suggests that screening for associated autoimmune conditions is unlikely to yield clinically meaningful results in JIA patients.

Overall, the utility of common lab tests like ESR in diagnosing and managing JIA remains limited. Continued research into genetic and immunological markers, such as HLA-B27, could enhance physicians' ability to anticipate more aggressive disease courses, identify risks for complications like uveitis, and optimize treatment plans.

## Conclusions

Given the absence of a singular diagnostic test for JIA, early diagnosis remains a significant challenge. Currently, diagnosis often requires an extensive list of differentials such as septic arthritis, other autoimmune conditions, joint fractures, lymphoproliferative disorders, and other possible conditions. While individually each lab test does not provide sufficient evidence for diagnosis by itself, only when combined, do these tests complement and reinforce each other’s findings, allowing physicians to make a more confident diagnosis. Although combining test findings can be effective, it is time-consuming, costly, and cannot replace a singular "gold standard" test which, unfortunately, is not currently available.

Given the concern for an underlying autoimmune condition, the patient was referred to a rheumatologist. Ultimately, this case serves as a reminder of the importance of continued monitoring and interdisciplinary collaboration when addressing complex musculoskeletal complaints in children.
